# Crossover from Jamming to Clogging Behaviours in Heterogeneous Environments

**DOI:** 10.1038/s41598-018-28256-6

**Published:** 2018-07-06

**Authors:** H. Péter, A. Libál, C. Reichhardt, C. J. O. Reichhardt

**Affiliations:** 1 Theoretical Division, Los Alamos National Laboratory, Los Alamos, NM 87545 USA; 2 Faculty of Mathematics and Computer Science, Babeş-Bolyai University, Cluj, 400084 Romania

## Abstract

Jamming describes a transition from a flowing or liquid state to a solid or rigid state in a loose assembly of particles such as grains or bubbles. In contrast, clogging describes the ceasing of the flow of particulate matter through a bottleneck. It is not clear how to distinguish jamming from clogging, nor is it known whether they are distinct phenomena or fundamentally the same. We examine an assembly of disks moving through a random obstacle array and identify a transition from clogging to jamming behavior as the disk density increases. The clogging transition has characteristics of an absorbing phase transition, with the disks evolving into a heterogeneous phase-separated clogged state after a critical diverging transient time. In contrast, jamming is a rapid process in which the disks form a homogeneous motionless packing, with a rigidity length scale that diverges as the jamming density is approached.

## Introduction

The concept of jamming is used in loose assemblies of particles such as grains or bubbles to describe the transition from an easily flowing fluidlike state to a rigid jammed or solidlike state^1–4^. Liu and Nagel proposed a generalized jamming phase diagram combining temperature, load, and density, where a particularly important point is the density *ϕ*_*j*_ at which jamming occurs^1^. Jamming has been extensively studied in a variety of systems^3–5^, and there is evidence that in certain cases, the jamming transition has the properties of a critical point, such as a correlation length that diverges as the jamming density is approached^2–9^. A related phenomenon is the clogging that occurs for particles flowing through a hopper, where as a function of time there is a probability for arch structures to form that block the flow^10–13^. Clogging is associated with the motion of particulate matter past a physical constraint such as wells, barriers, obstacles, or bottlenecks^14–19^; however, it has not been established whether jamming and clogging are two forms of the same phenomenon or whether there are key features that distinguish jamming from clogging.

Here we show for frictionless disks moving through a random obstacle array that jamming and clogging are distinct phenomena and that a transition from clogging to jamming occurs as a function of increasing disk density. We identify the number of obstacles required to stop the flow and the transient times needed to reach a stationary clogged or jammed state. There are two critical obstacle densities, ${\phi }_{c}^{j}$ for the jammed state and ${\phi }_{c}^{c}$ for the clogged state. In the jamming regime, the obstacle density ${\phi }_{c}^{j}$ at which flow ceases drops to lower obstacle densities with increasing disk density, and the system forms a homogeneous jammed state when the rigidity correlation length associated with *ϕ*_*j*_ becomes larger than the average distance between obstacles. In contrast, during clogging the system organizes over time into a heterogeneous or phase-separated state, and the transient time diverges at a critical obstacle density ${\phi }_{c}^{c}$ that is independent of the disk density. The phase-separated state consists of regions with a density near *ϕ*_*j*_ coexisting with low density regions.

## Results

### Time evolution to a jammed or clogged state

**Figure 1:**
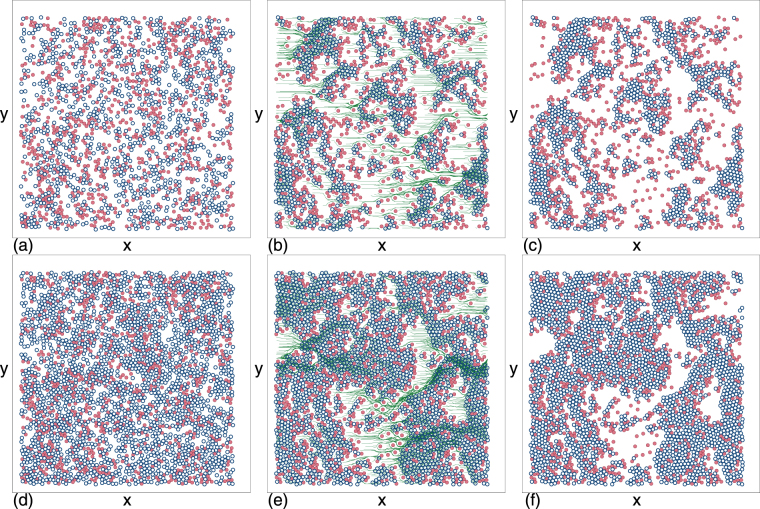
Clogging in obstacle arrays. Images of (**a**,**d**) initial, (**b**,**e**) transient flowing, and (**c**,**f**) final clogged state for mobile disks (dark blue open circles) driven in the positive *x* direction through obstacles (red filled circles) in a sample with obstacle density *ϕ*_*obs*_ = 0.175 and disk density (**a**–**c**) *ϕ*_*m*_ = 0.2186 and (**d**–**f**) *ϕ*_*m*_ = 0.436. Green lines in (**b**,**e**) indicate the disk trajectories over a fixed time period. The disks are initially in a flowing state and evolve into a phase-separated clogged state. The dense regions have a local disk density of *ϕ*_loc_ = 0.84.

**Figure 2:**
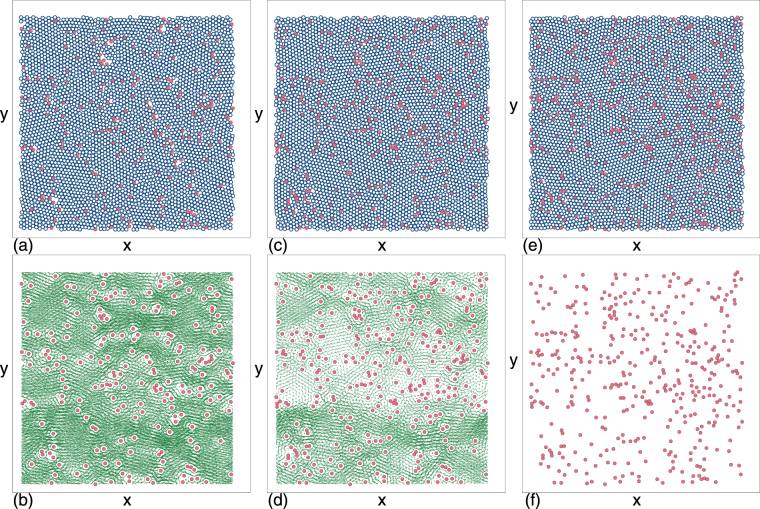
Jamming in obstacle arrays under increasing obstacle density in a sample with *ϕ*_*m*_ = 0.785. The randomly placed obstacles prevent the monodisperse disks from forming a state with crystalline ordering. (**a**,**c**,**e**) Mobile disks (dark blue open circles) and obstacles (red filled circles). (**b**,**d**,**f**) Corresponding disk trajectories over a fixed time period (green lines) and obstacle locations (red filled circles), with the mobile disks omitted for clarity. (**a**,**b**) At an obstacle density of *ϕ*_*obs*_ = 0.043, we find steady state flow. (**c**,**d**) At *ϕ*_*obs*_ = 0.065 the steady state flow is reduced but still present. (**e**,**f**) At *ϕ*_*obs*_ = 0.0872, the system jams. The jammed state is much more uniform in density than the clogged state, and jamming occurs rapidly with almost no transient flow above a critical obstacle density ${\phi }_{c}^{j}$.

We numerically examine disks driven through a two-dimensional array of obstacles in the form of immobile disks. Simulation details appear in the Methods section. The total area density of the system is *ϕ*_tot_ = *ϕ*_*m*_ + *ϕ*_*obs*_, where *ϕ*_*m*_ is the area density of the moving disks and *ϕ*_*obs*_ is the area density of the obstacles. Starting from a uniformly dense sample, we apply a driving force and find that over time the system evolves either to a steady free flowing state or to a motionless clogged or jammed state. In Fig. 1a–c we illustrate the time evolution of a system with a disk density of *ϕ*_*m*_ = 0.2186 and an obstacle density of *ϕ*_*obs*_ = 0.175, beginning with the uniform density initial state in Fig. 1a. Upon application of a drive, we find a transient flowing state as shown in Fig. 1b which gradually evolves into the final motionless phase-separated or clustered clogged state in Fig. 1c. For a higher disk density of *ϕ*_*m*_ = 0.436, Fig. 1d–f shows that the same evolution from uniform initial state to transient flowing state to static clogged state occurs, but the dense clusters in the clogged state are larger. At much higher disk densities of *ϕ*_*m*_ = 0.785, we find jamming behavior when the obstacle density is larger than a critical value ${\phi }_{c}^{j}$. Below ${\phi }_{c}^{j}$, the system quickly settles into steady state flow, as shown in Fig. 2(a,b) for *ϕ*_*obs*_ = 0.043 and in Fig. 2(c,d) for *ϕ*_*obs*_ = 0.065. The magnitude of the flow decreases with increasing *ϕ*_*obs*_. Above ${\phi }_{c}^{j}$ the disks quickly form a disordered jammed state when driven, as illustrated in Fig. 2(e,f) for *ϕ*_*obs*_ = 0.0872. In contrast to the density phase-separated clogged states that form at lower *ϕ*_*m*_, the jammed states are homogeneously dense. The randomly placed obstacles prevent the monodisperse disks from developing long-range crystalline order.

### Velocity measurement of the transition from clogging to jamming

**Figure 3:**
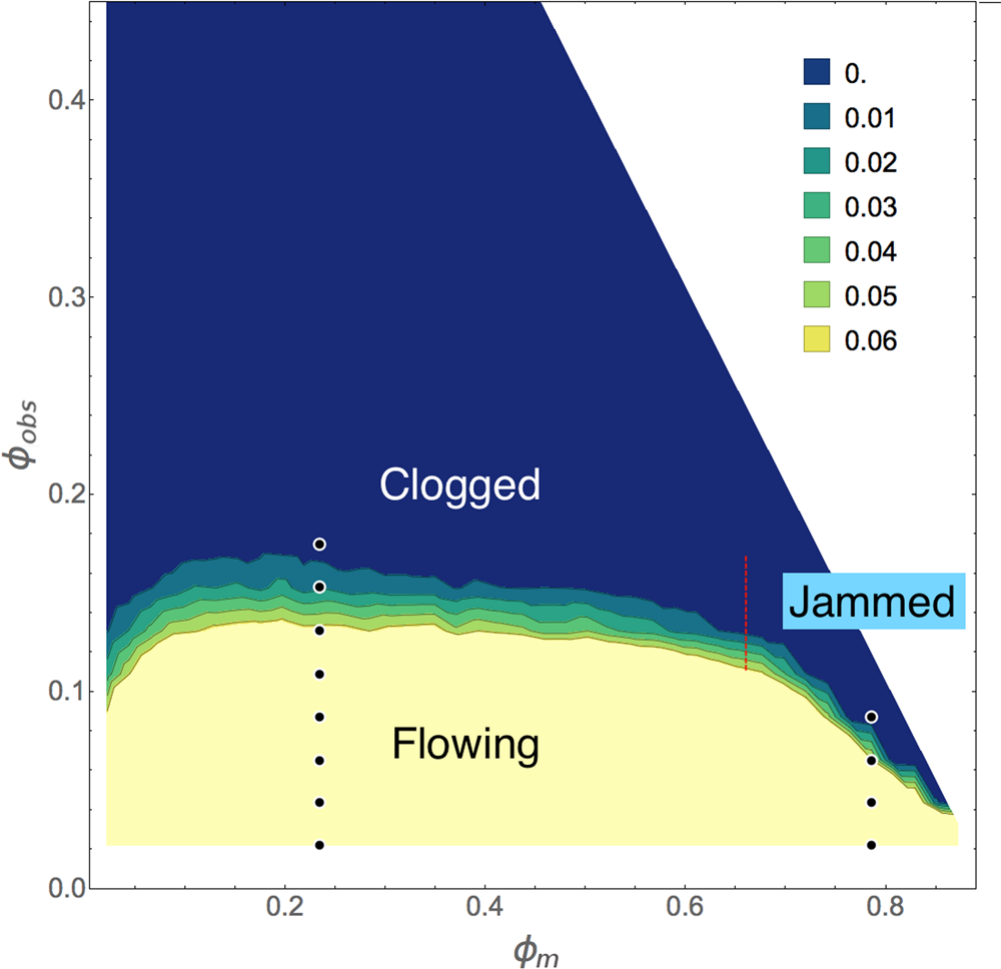
Clogging-jamming phase diagram. The heat map of the disk velocity *V*_0_ after 10^6^ simulation time steps as a function of obstacle density *ϕ*_*obs*_ vs disk density *ϕ*_*m*_. Yellow indicates high *V*_0_ and blue indicates zero *V*_0_. The white region at the upper right is above the density ${\phi }_{{\rm{tot}}}=\pi /2\sqrt{3}\approx 0.9069$ at which the disks would form a hexagonal solid, and thus lies outside the range of our model. Clogging occurs for *ϕ*_*m*_ < 0.67, and the critical obstacle density for clogging ${\phi }_{c}^{c}\approx 0.15$, is nearly independent of *ϕ*_*m*_. Jamming occurs for *ϕ*_*m*_ > 0.67, as indicated by the red vertical dashed line, and ${\phi }_{c}^{j}$, the critical obstacle density for jamming, decreases linearly with increasing *ϕ*_*m*_, ranging from $0.15 > {\phi }_{c}^{j} > 0$. The dots along *ϕ*_*m*_ = 0.234 indicate the values of *ϕ*_*obs*_ shown in the time series of Fig. 4a, while the dots along *ϕ*_*m*_ = 0.785 indicate the values of *ϕ*_*obs*_ shown in the time series of Fig. 4b.

To characterize the system we perform a series of simulations with varied *ϕ*_*m*_ and *ϕ*_*obs*_. We measure the final velocity *V*_0_ of the mobile disks after a fixed time interval, and average over ten different realizations. In Fig. 3 we plot a velocity heat map as a function of *ϕ*_*obs*_ versus *ϕ*_*m*_. We find a flowing regime at small *ϕ*_*obs*_, a clogged regime for *ϕ*_*m*_ < 0.67, and a jammed regime for *ϕ*_*m*_ > 0.67. The critical obstacle density ${\phi }_{c}^{c}$ above which the velocity *V*_0_ drops to zero in the clogging regime remains roughly constant at ${\phi }_{c}^{c}\approx 0.15$, independent of the value of *ϕ*_*m*_. This indicates that the transition to a clogged state is controlled by the average spacing ${l}_{obs}=1/\sqrt{{\phi }_{obs}}$ between obstacles, similar to the manner in which hopper clogging is controlled by the aperture size. In contrast, in the jamming regime the critical obstacle density ${\phi }_{c}^{j}$ separating flowing from jammed states decreases linearly with increasing *ϕ*_*m*_ and reaches ${\phi }_{c}^{j}=0$ for *ϕ*_*m*_ ≈ 0.9069, indicating that this transition is controlled by a growing correlation length *ξ* associated with the jamming point *ϕ*_*j*_^5^. We argue that the system jams when *ξ* = *l*_*obs*_. If we assume that near jamming in a clean system, the correlation length grows as *ξ* ∝ (*ϕ*_*j*_ − *ϕ*_*m*_)^−*ν*^, then the transition to the jammed state varies with obstacle density according to ${\phi }_{c}^{j}\propto {({\phi }_{j}-{\phi }_{m})}^{2\nu }$. In Fig. 3, ${\phi }_{c}^{j}\propto {\phi }_{m}$, implying that *ν* = 1/2, consistent with the exponent *ν* = 1/2 proposed for jamming in refs.^20,21^, as well as with simulation measurements giving *ν* in the range 0.6 to 0.7 for two-dimensional bidisperse disks^6,8^. The exponent we find is also in agreement with that observed for the shift in the jamming point in bidisperse disks on random pinning arrays^22^. Studies of bidisperse disk jamming with dilute obstacles very near *ϕ*_*j*_ also show that *ϕ*_*j*_ decreases linearly with obstacle density, giving *ν* = 1/2^23^.

Previous simulations of bidisperse disks of radius *R*_*s*_ = 0.5 and *R*_*l*_ = 0.7 flowing through a periodic array of obstacles with radius *R*_*s*_ = 0.5 showed that clogging is strongly enhanced when ${l}_{obs}\lesssim 2.35$^24^. This is because in order for a pair of disks, one large and one small, to fit between two obstacles, the lattice constant *a* of the obstacle array must be at least large enough to accommodate the size of the obstacle itself plus the size of the two disks, *a* ≥ 2*R*_*s*_ + 2*R*_*s*_ + 2*R*_*l*_ = 2.4. In our monodisperse disk system, the obstacles are placed randomly, but one can obtain an estimate of the *l*_*obs*_ for the onset of clogging by considering the circular holes in the obstacle array^25^. If we construct a circle that just touches any three disks and/or obstacles, this circle is defined to be a hole when it does not overlap any disks. For a pair of disks to pass between two obstacles, the obstacle spacing must once again accommodate the size of the obstacle itself plus the size of the two disks, ${l}_{obs}\ge {l}_{obs}^{c}=2{R}_{d}+2{R}_{d}+2{R}_{d}=3.0$. This spacing can be achieved by placing the obstacles such that circular holes of size *R*_*d*_ can form on all sides of the hole on average, giving an effective obstacle radius of 3*R*_*d*_ and a critical obstacle density of ${\phi }_{obs}^{c}=(1/6)\,(\pi /(2\sqrt{3}))=0.15$. Below ${\phi }_{obs}^{c}$, the holes percolate and the disks can flow, while above ${\phi }_{obs}^{c}$, not enough holes are available to permit steady state free flow and a clogged state forms. In Fig. 3, the onset of clogging, ${\phi }_{c}^{c}\approx 0.15$, is close to ${\phi }_{obs}^{c}$. When ${\phi }_{m}\lesssim 0.15$, ${\phi }_{c}^{c}$ is no longer constant but decreases with decreasing *ϕ*_*m*_. At these low disk densities, mobile disks are trapped independently, so at least one additional obstacle must be added for every additional mobile disk, giving ${\phi }_{c}^{c}\propto {\phi }_{m}$.

### Transient velocities near the clogging and jamming transitions

**Figure 4:**
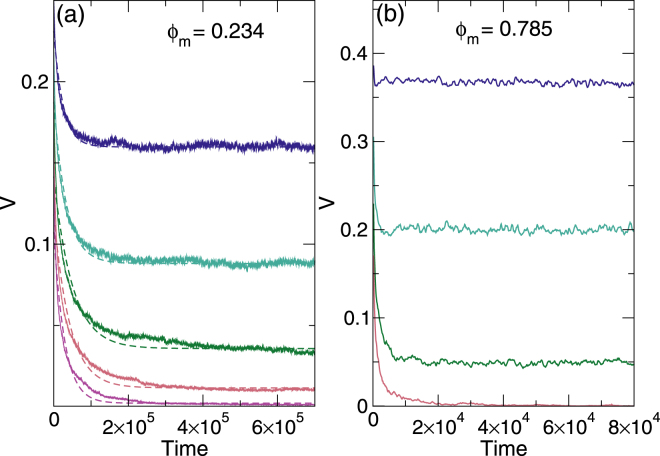
Transient times for clogging and jamming. (**a**) The average velocity *V* per mobile disk vs time in simulation time steps for samples with mobile disk density *ϕ*_*m*_ = 0.234 at varied obstacle density *ϕ*_*obs*_ = 0.087, 0.109, 0.131, 0.153, and 0.175, from top to bottom. Dashed lines indicate fits to $V\propto A\,\exp (\,-\,t/\tau )+{V}_{0}$. The disks reach a clogged state for *ϕ*_*obs*_ > 0.15. (**b**) *V* vs time for samples with *ϕ*_*m*_ = 0.785 in the jamming regime for *ϕ*_*obs*_ = 0.022, 0.044, 0.065, and 0.087, from top to bottom. The transient times are very short.

**Figure 5:**
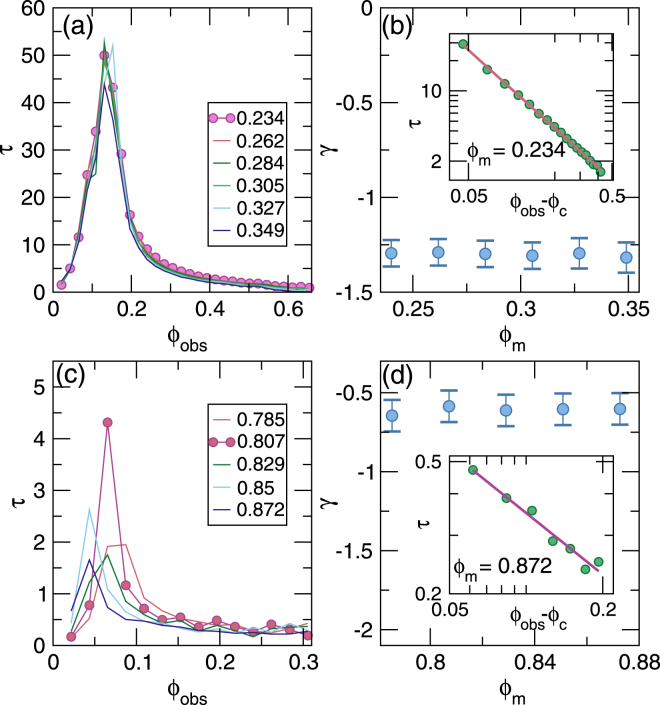
Transient times for clogging and jamming. (**a**) Transient times *τ* vs *ϕ*_*obs*_ obtained from *V*(*t*) curves such as those shown in Fig. 4a by fitting $V\propto A\,\exp (\,-\,t/\tau )+{V}_{0}$ for *ϕ*_*m*_ = 0.234 to 0.349, from top to bottom. There is a divergence in *τ* near the clogging density of ${\phi }_{c}^{c}=0.15$. (**b**) The value of the exponent *γ* vs *ϕ*_*m*_ obtained by fitting the curves in **a** to $\tau \propto {({\phi }_{obs}-{\phi }_{c}^{c})}^{\gamma }$. Inset: *τ* vs ${\phi }_{obs}-{\phi }_{c}^{c}$ at *ϕ*_*m*_ = 0.234. The pink line indicates a power law fit with *γ* = −1.29 ± 0.1. (**c**) *τ* vs *ϕ*_*obs*_ obtained from the *V*(*t*) curves such as those shown in Fig. 4b for *ϕ*_*m*_ = 0.785 to 0.872, from top to bottom. The transient times are much shorter than those in the clogging regime in panel (a). (**d**) Exponent *γ* vs *ϕ*_*m*_ obtained by fitting the curves in (**c**) to $\tau \propto {({\phi }_{obs}-{\phi }_{c}^{j})}^{\gamma }$. Inset: *τ* vs ${\phi }_{obs}-{\phi }_{c}^{j}$ at *ϕ*_*m*_ = 0.872. The pink line indicates a power law fit with *γ* = −0.66.

In Fig. 4a we show representative time series of the average velocity *V* per mobile disk in the clogging regime at *ϕ*_*m*_ = 0.234 for *ϕ*_*obs*_ ranging from *ϕ*_*obs*_ = 0.087 to 0.175. At low obstacle densities, the disks reach a steady state flow after a very short transient time *τ*. As *ϕ*_*obs*_ increases, *τ* increases, showing a divergence at the critical obstacle density ${\phi }_{c}^{c}$ where clogging first occurs, while for ${\phi }_{obs} > {\phi }_{c}^{c}$, *τ* decreases with increasing *ϕ*_*obs*_ and the disks reach a completely clogged state with *V* = 0. We fit $V(t)\propto A\,\exp (\,-\,t/\tau )+{V}_{0}$, as indicated by the dashed lines in Fig. 4(a), and plot the resulting values of *τ* in Fig. 5a as a function of *ϕ*_*obs*_ for *ϕ*_*m*_ = 0.234 to 0.349. In each case, *τ* diverges near *ϕ*_*obs*_ = 0.15. We fit this divergence for ${\phi }_{obs} > {\phi }_{c}^{c}$ to a power law, $\tau \propto {({\phi }_{obs}-{\phi }_{c}^{c})}^{\gamma }$, as shown in the inset of Fig. 5b for *ϕ*_*m*_ = 0.234, where *γ* = −1.29 ± 0.1. The plot of *γ* versus *ϕ*_*m*_ in the main panel of Fig. 5(b) indicates that *γ* has a constant value in the range −1.25 to −1.35. The transient time behavior is similar to that found for the diverging time scales that appear near the irreversible-reversible transition in systems exhibiting random organization^26–28^ and near the depinning transition for colloids^29^ and vortices^30,31^ driven over random pinning arrays. The power law exponents are also close to the value *γ* = −1.295 expected for the universality class of two-dimensional directed percolation^32^, and we find similar values of *γ* for *ϕ*_*m*_ < 0.67 throughout the clogging regime. Directed percolation is often used to describe nonequilibrium absorbing phase transitions^32^, and in our case the steady state flow corresponds to a fluctuating state, while the clogged state is the non-fluctuating or absorbed state.

In the jamming regime the transient times are much shorter, as shown by the plot of *V*(*t*) in Fig. 4b for *ϕ*_*m*_ = 0.785 at *ϕ*_obs_ = 0.022 to 0.087. In Fig. 5c, *τ* versus *ϕ*_*obs*_ in the range *ϕ*_*m*_ = 0.785 to 0.872 has a value that is an average of 20 times smaller than in the clogging regime from Fig. 5a. The peak in *τ* shifts to lower *ϕ*_*obs*_ with increasing *ϕ*_*m*_, reflecting the behavior of the critical jamming density ${\phi }_{c}^{j}$. By fitting the curves in Fig. 5c to $\tau \propto {({\phi }_{obs}-{\phi }_{c}^{j})}^{\gamma }$, as demonstrated in the inset of Fig. 5d for *ϕ*_*m*_ = 0.872, we obtain *γ* ≈ −0.66, as shown in the plot of *γ* versus *ϕ*_*m*_ in the main panel of Fig. 5d. This indicates that there is a pronounced difference in the dynamics of the jamming regime compared to the clogging regime.

**Figure 6:**
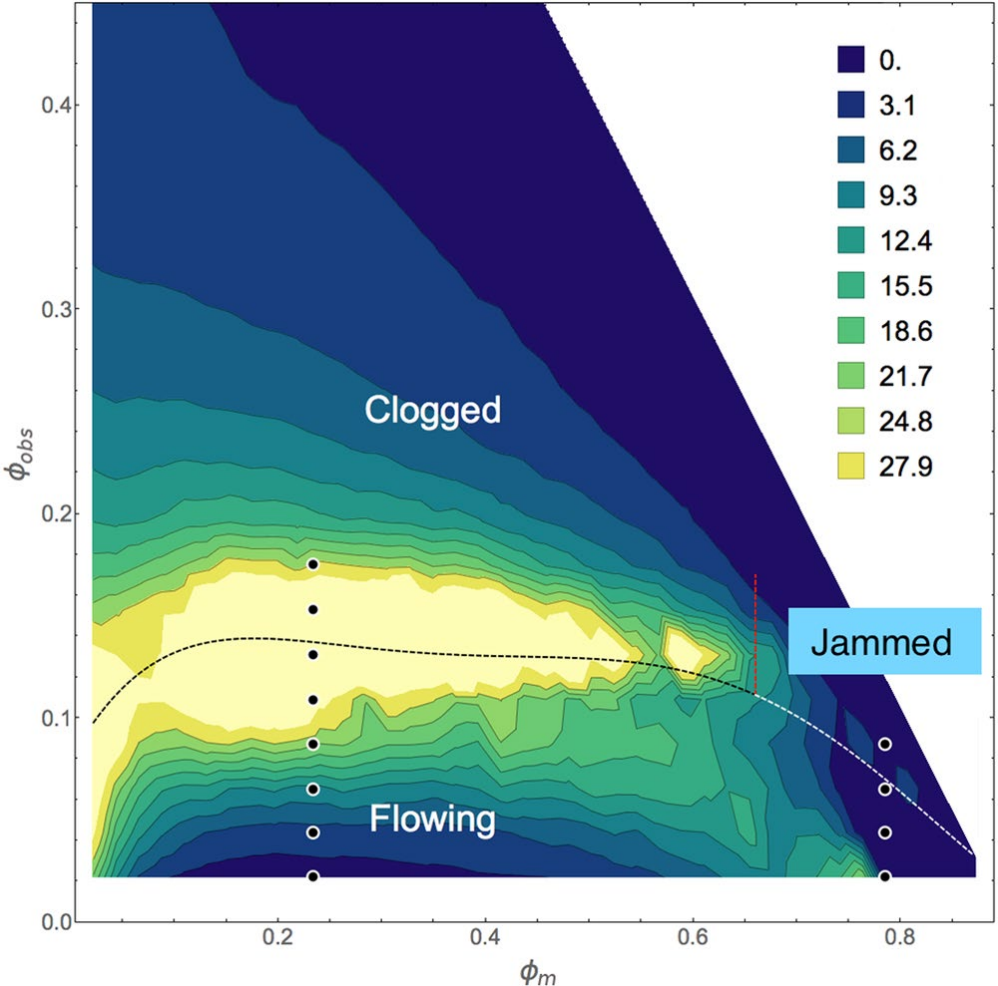
Transient time behavior. The heat map of the transient times *τ* obtained from fitting $V(t)=A\,\exp (\,-\,t/\tau )+{V}_{0}$ as a function of *ϕ*_*obs*_ vs *ϕ*_*m*_. Yellow indicates large *τ* and blue indicates small *τ*. The dark dashed line is a guide to the eye marking the crossover from a flowing state to a clogged state, while the white dashed line indicates the transition from a flowing state to a jammed state. The white region is above the maximum density ${\phi }_{{\rm{tot}}}=\pi /2\sqrt{3}$ of our model. The dots along *ϕ*_*m*_ = 0.234 indicate the values of *ϕ*_*obs*_ shown in the time series of Fig. 4a, while the dots along *ϕ*_*m*_ = 0.785 indicate the values of *ϕ*_*obs*_ shown in the time series of Fig. 4b. The system must organize into a clogged state, giving large transient times in the clogging regime, but can quickly enter a jammed state, giving small transient times in the jamming regime.

In Fig. 6 we show a heat map of the transient time *τ* obtained by fitting $V(t)=A\,\exp (\,-\,t/\tau )+{V}_{0}$. The transient times become large near the crossover from flowing to clogging for *ϕ*_*m*_ < 0.67, while in the jamming regime for *ϕ*_*m*_ > 0.67, the transient times are strongly reduced. This provides further evidence that in the clogging regime it is necessary for the system to organize over time into a clogged state, gradually forming phase-separated regions of high and low density as illustrated in Fig. 1. In contrast, the jammed system has strong spatial correlations, and once the correlation length associated with *ϕ*_*j*_ is larger than the distance *l*_*obs*_ between defects, very few disk rearrangements are needed to bring the system into a stationary, nonflowing state.

**Figure 7:**
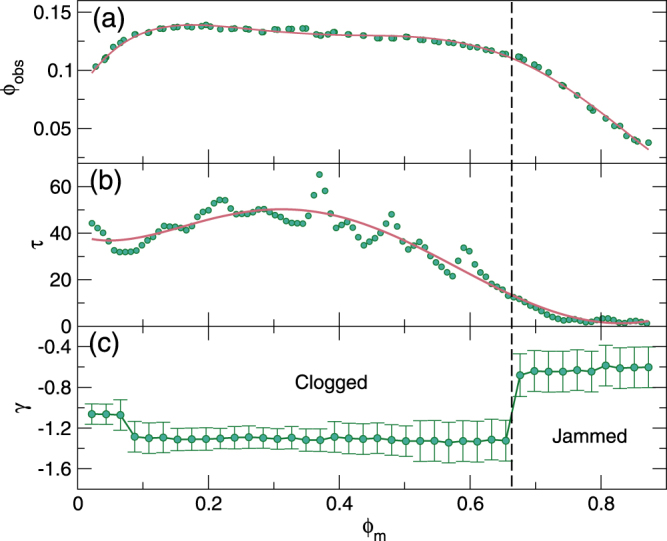
Transient times and critical exponents across the clogging to jamming transition. (**a**) The location of the transition from a flowing state to a clogged or jammed state, defined as points for which *V*_0_ = 0.01, as a function of *ϕ*_*obs*_ vs *ϕ*_*m*_. The dashed line separates clogged states at low *ϕ*_*m*_ from jammed states at high *ϕ*_*m*_. (**b**) The transient time *τ* at the flowing to nonflowing transition point vs *ϕ*_*m*_. (**c**) The transient exponent *γ* extracted from the nonflowing side of the transition vs *ϕ*_*m*_. There is a clear crossover from clogging to jamming. In the clogging regime, *γ* ≈ −1.29, but in the jamming regime, *γ* ≈ −0.66, indicating that the dynamics of clogging differ from those of jamming.

In Fig. 7a we show the transition from the flowing to the clogged or jammed state as a function of *ϕ*_*obs*_ versus *ϕ*_*m*_ by identifying the points from Fig. 3 for which *V* = 0.01. Figure 7b shows the transient times *τ* along this transition line, and in Fig. 7c we plot the transient exponent *γ*. The dashed vertical line at *ϕ*_*m*_ = 0.67 indicates a transition from clogging to jamming behavior, correlated with a change from *γ* ≈ −1.29 in the clogging regime to *γ* ≈ −0.66 in the jamming regime, as well as with a drop in *ϕ*_*obs*_ and *τ*. The point *ϕ*_*m*_ = 0.67 matches the density at which 2D continuum percolation of disks is expected to occur. We find a third value of *γ* for *ϕ*_*m*_ < 0.07 in a density regime where the value of *ϕ*_*obs*_ at which a clogged state appears decreases with decreasing *ϕ*_*m*_. This regime is dominated by the trapping of single disks rather than collective clogging dynamics.

### Local disk densities in clogged and jammed states

**Figure 8:**
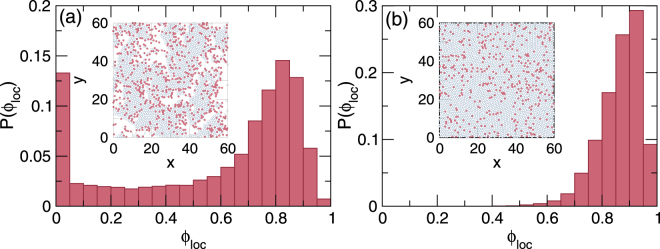
Local density distributions in clogged and jammed systems. (**a**) The local density distribution *P*(*ϕ*_loc_) averaged over 10 realizations for a system with *ϕ*_*m*_ = 0.5 and *ϕ*_*obs*_ = 0.175 that reaches a clogged state in which the local density is bimodally distributed. Inset: Image of a clogged configuration from one of the realizations, showing the mobile disks (dark blue open circles) trapped by the obstacles (red filled circles). (**b**) *P*(*ϕ*_loc_) averaged over ten realizations for a jammed system with *ϕ*_*m*_ = 0.8 and *ϕ*_*obs*_ = 0.06 shows a single peak at *ϕ*_tot_. Inset: Image of a jammed disordered configuration from one of the realizations.

The clogged and jammed systems can also be distinguished by examining the local disk density *ϕ*_loc_ measured in areas 6*R*_*d*_ × 6*R*_*d*_ in size. In Fig. 8a we plot the local density distribution *P*(*ϕ*_loc_) averaged over ten realizations of the final clogged state for a system with *ϕ*_*m*_ = 0.5 and *ϕ*_*obs*_ = 0.175. As shown in the inset of Fig. 8a, the disks phase separate into low density regions associated with the peak at *ϕ*_loc_ = 0.1 and high density regions which produce a second peak at *ϕ*_loc_ = 0.85. The local density of the dense regions is lower than the value of *ϕ*_loc_ = 0.9069 for a dense ordered hexagonal disk arrangement due to the considerable disorder introduced in the packing by the randomly placed obstacles. In Fig. 8b, *P*(*ϕ*_loc_) for a system with *ϕ*_*m*_ = 0.8 and *ϕ*_*obs*_ = 0.06 that reaches a jammed state has a single peak near *ϕ*_loc_ = 0.9, reflecting the uniform disk density at jamming that is illustrated in the inset of Fig. 8(b).

## Discussion

Our results suggest that clogging and jamming processes have different dynamics. Clogging in the presence of random obstacles has signatures of an absorbing transition falling in a directed percolation universality class, and its dynamics are controlled by the average spacing of the obstacles. In the jamming that occurs for higher *ϕ*_tot_, the dynamics are controlled by the growing correlation length associated with *ϕ*_*j*_, the jamming density of an obstacle-free system. These results show that jamming and clogging in obstacles are indeed different phenomena. Jamming is associated with an equilibrium critical point, the formation of a homogeneous rigid state, and short transient times to reach this state, while clogging is a nonequilibrium dynamical phenomenon in which the system evolves over an extended time into a strongly spatially heterogeneous state. Our results have implications for flow though heterogeneous media^33^, erosion^34^, depinning transitions in particle assemblies^35^, and active matter in disordered environments^36,37^. Experimentally our results could be tested using colloidal particles at low flow rates to reduce hydrodynamic effects. It would also be interesting to examine the effects of adding frictional contacts between the disks, since these can change the characteristics of the jamming transition^38,39^, or to replace the disks by elongated particles^40^ or chains^41,42^.

## Methods

### Numerical simulation details

We conduct simulations of nonoverlapping disks and obstacles confined to a two-dimensional plane. The system size is *L* × *L* with *L* = 60, and we use periodic boundary conditions in both the *x* and *y* directions. We introduce *N*_*m*_ mobile disks of radius *R*_*d*_ = 0.5 along with *N*_*obs*_ obstacles represented by disks of radius *R*_*d*_ that are not allowed to move. The area coverage of the mobile disks is ${\phi }_{m}={N}_{m}\pi {R}_{d}^{2}/{L}^{2}$, the area coverage of the obstacles is ${\phi }_{obs}={N}_{{ob}{s}}\pi {R}_{d}^{2}/{L}^{2}$, and the total area coverage is *ϕ*_tot_ = *ϕ*_*m*_ + *ϕ*_*obs*_.

The disk dynamics are given by the overdamped equation of motion

1 $$\frac{1}{\eta }\frac{{\rm{\Delta }}{{\bf{r}}}_{i}}{{\rm{\Delta }}t}={{\bf{F}}}_{pp}^{i}+{{\bf{F}}}_{{\rm{obs}}}^{i}+{{\bf{F}}}_{d}$$

where *η* = 1 is the viscosity. The interaction between two disks at **r**_*i*_ and **r**_*j*_ is a short range harmonic repulsion, ${{\bf{F}}}_{dd}^{ij}=k({r}_{ij}-2{R}_{d})\,{\rm{\Theta }}({r}_{ij}-2{R}_{d}){\hat{{\bf{r}}}}_{ij}$, where *r*_*ij*_ = |**r**_*i*_ − **r**_*j*_|, ${\hat{{\bf{r}}}}_{ij}=({{\bf{r}}}_{i}-{{\bf{r}}}_{j})/{r}_{ij}$, and Θ is the Heaviside step function. We set *k* = 200, which is large enough that overlap between disks does not exceed 0.01*R*_*d*_, placing us in the hard disk limit. The interactions with mobile disks are given by ${{\bf{F}}}_{pp}^{i}={\sum }_{j\ne i}^{{N}_{p}}\,{{\bf{F}}}_{dd}^{ij}$, while the interactions with obstacles are given by ${{\bf{F}}}_{{\rm{obs}}}^{i}={\sum }_{j}^{{N}_{ps}}\,{{\bf{F}}}_{dd}^{ij}$. We apply a uniform driving force ${{\bf{F}}}_{d}={F}_{d}\hat{{\bf{x}}}$ to all mobile disks, with *F*_*d*_ = 0.5. Distances are measured in simulation units *l*_0_ and forces are measured in simulation units *f*_0_ so that *k* is in units of *f*_0_/*l*_0_ and the unit of simulation time is *t*_0_ = *ηl*_0_/*f*_0_. We initialize the system by placing *N*_*m*_ + *N*_*obs*_ disks of reduced radius in randomly chosen nonoverlapping positions, and then gradually expanding the radii to size *R*_*d*_ while allowing all disks to move. This produces a randomized packing of homogeneous density with no internal tensions. We then randomly assign *N*_*obs*_ of the disks to be obstacles, and apply an external driving force. After a fixed simulation time of 1 × 10^6^ simulation time steps, we determine whether the system has reached a clogged or jammed state based on whether the average disk velocity *V* has dropped to zero.
